# Comparative Pathology

**Published:** 1894-12

**Authors:** A. J. Harrison

**Affiliations:** Physician to the Bristol General Hospital, and Lecturer on Medical Jurisprudence in University College, Bristol


THE BRISTOL

Medico-Chirurgical Journal.

DECEMBER, 1894.

### COMPARATIVE PATHOLOGY.

### A STUDY IN THE GARDENS OF THE CLIFTON ZOOLOGICAL SOCIETY.

The Presidential Address, delivered on October 10th, 1894, at the opening of the 21st Session of the Bristol Medico-Chirurgical Society.

BY

A. J. HARRISON, M.B. Lond.,

*Physician to the Bristol General Hospital, and Lecturer on Medical Jurisprudence in University College, Bristol.*

I CAN assure you, I appreciate very highly indeed the honour you have conferred upon me in electing me the President of this very important and excellent Society; certainly, to praise it in the lower strain, the most important medico-scientific Society in the whole of the West of England, and had I not your goodwill and wishes to encourage me, I might well feel appalled to occupy the presidential chair this evening, when I reflect upon the stirring and clever addresses which adorn the archives of the Bristol Medico-Chirurgical Society.

In anticipation I, naturally, have passed many subjects through my mind in order to arrive at one which might be acceptable. We have had glorious reminiscences of the past, sanguine forecasts of the future, the medical essences and psychological problems of Shakspere produced in a most fascinating manner, records of mistakes, shortcomings, and repentances,—and would that these were more frequent, for more is sometimes learnt from failures and disappointments than from long parades of successes; then, only last year, we had a most eloquent discourse on books, what they are and what they ought to be, and on the weaknesses of editors in pandering to the depravity of literary tastes.

At one time I thought of putting together my professional experience—of now exactly forty years' duration. Its early details would have embraced the time when chloroform was first being generally used, and the stethoscope had hardly established itself as a general appliance; when hospitals were much fewer in the country, and there were very few local hospitals and no cottage ones, and when patients suffering from fearful injuries—accidents by machinery, or extensive burns,—or from severe pneumonia, fever, bronchitis, phthisis, aneurism, &c., &c., were often conveyed many weary miles in country carts and wagons; and when burns and wounds generally, if dressed at all antiseptically, were done so rather by fortuitous accident than by intelligent design.

Or, I might have taken up the subject of Small-pox, and the to-day burning question of Vaccination. I could have related cases of the very worst forms—virulent, confluent, hemorrhagic—and have drawn comparisons between the past and the present, greatly in favour of the latter.

However, these subjects I have thought of and dismissed, not because they were not good and adequate ones, but because in this annual address each occupier of this chair takes up some theme which is nearer to him, and more in accordance with his tastes—or, in other words, he rides his "hobby" for the time being, and "struts and frets his hour upon the stage" in his most suitable character, as he may think, and in which he trusts he may be the most presentable; and therefore I claim your considerate indulgence whilst I conduct you,—in a somewhat discursive and fragmentary manner, I admit—along a pathological ramble, through our justly celebrated Zoological Gardens.

It is known to most of you that I have been connected with the Gardens for a good many years, and I have, during that time, come across and collected together a few interesting and, of necessity, somewhat rare details. They must be fragmentary, and therefore disjointed. I cannot undertake to arrange them in any anatomical, physiological, or even pathological order. These more scientific conditions can only be obtained after and through experiences far transcending mine. I only hope to make a few contributions towards such desirable records, since Comparative Pathology must throw some light upon human diseases, and will in the future form an important subject.

The first case was a very remarkable one, and, as far as I know, unique in many of its circumstances. Some years ago our Zoological Society possessed a very splendid specimen of the African lion, which rejoiced in the name of Hannibal. He was truly Hannibal I., for there has been a successor, the second of that renowned name. He was the pride of the Gardens, as he was held to be the finest lion in Europe. He always seemed in splendid condition, and until a few months before his death had never been ill. On the morning of April 29th, 1878, this grand specimen of the feline race was found dead in his cage. The underkeeper unlocked the house and passed his den, and happened to notice, as he thought, the animal asleep, his head resting on his forepaws. Returning in a few minutes, the man observed the lion in the same position; his suspicions were aroused, and he soon discovered the lion was stiff and dead.

An hour or two afterwards I saw Hannibal in the slaughter-yard at the back of the lion-house, and never shall I forget the impression produced by my first *dead* lion. His enormous limbs and magnificent proportions filled me with awe, and as I gazed I almost shuddered at the thought of the ease with which those massive and sinewy paws could have crushed me only a little while before; but verily, a live dog is better than a dead lion. His race was run, his seventeen years had been told. On opening the abdominal cavity, there was seen an enormous extravasation of blood, which had come from a ruptured spleen.

This organ weighed 21½ lbs. I afterwards endeavoured to ascertain the weight of the spleen in these large carnivora, and I have known it only nine ounces in a fairly healthy animal, and rarely, in any other case within my knowledge, coming up to a pound. It evidently is not a large organ in Felidæ. The heart and valves seemed quite healthy; there was a small clot in the left ventricle, and a little dark blood in the right auricle. The lungs were congested at both bases, and the right one had a carnified mass in it as large as a small orange—probably a hemorrhagic infarct of oldish date, or a result of inflammation. The kidneys, which were a good deal congested, seemed large, but I cannot give comparative weights. The liver was large, and contained a cystic tumour in the anterior part of the large lobe, near the gall-bladder. The cyst contained clear, colourless fluid. The stomach and intestines were apparently quite healthy. The stomach contained a quantity of mucus—scarcely acid to litmus paper; its muscular coats, and those of the small gut, were thick and well-developed. The splenic enlargement seemed to be caused by hyperæmia and increase in the lymphatic and vascular elements—but what was the etiology? Are lions subject to malarious attacks? and had Hannibal been a victim in the days of his youth, in his native wilds—for he was forest-bred—before the civilization of captivity had fallen upon him? He had been ill a couple of months or so before his death, when his breathing was affected. Did he have pneumonia then, with carnification of the base of the right lung—or perhaps more probably a hemorrhage from an embolism,—or are lions subject to splenic fever?

There remained no doubt that death had been caused by hemorrhage arising from rupture of the spleen; and the following is, doubtless, the explanation. The lion had copulated with the lioness on the previous day at 10 a.m. and again at 6 p.m. There was no eye-witness of these acts, but the head-keeper, Blunsden, who is an experienced and observant man, said he felt certain of the fact because he had heard the lion make a very peculiar roar at those times; and he added, "I should know what that roar meant if I heard it a mile away." The sequel proved that he was correct. The animal during the evening act of copulation had ruptured his enormously enlarged spleen, and then had gradually, but inevitably, bled to death during the night. His huge spleen had not caused "desire to fail" and consummation of the act or acts was accomplished, and resulted in the lioness (kept alone during this period of her widowhood) producing in due time three fine cubs—two males and a female. One of them grew into the celebrated Jupiter, which we shall hear about again later on; the female grew up and was sold; and the third died on June 16th, 1881, from a kind of distemper of long duration, and accompanied with skin disease. I said the cubs were born in due time. What is this due time? We were anxious to verify the head-keeper's opinion that the period of gestation was sixteen weeks, and here was an opportunity. From the 29th of April to August 18th constituted sixteen weeks: the cubs were born on August 16th.[1]

There are tragedies enacted in the "Zoo." Listen to a select few.

On the same day that I made the *post-mortem* examination of Hannibal, a small monkey had been humanely killed because its companions had gnawed one of its arms off. The limb had somehow got entangled in the wires of the cage, and in their wisdom the other monkeys seem to have thought this mode of extrication the most ready one. Perhaps ardent admirers of "Darwinism" may in this recognise advance of type—rudimentary surgery amongst the "caricatures of man." I cannot report as yet any progress in this direction; but then the steps are very slow, and the time since has been short. The viscera of this sacrifice to surgery seemed quite healthy; but what struck me as remarkable then—and it has been corroborated since by many observations—was this, the very small ratio of *lung tissue* the quadrumana have. The lungs of monkeys seem to me to correspond very much with what we see about their antics—a succession of little movements, quick and often, a leap, a

[1] Lions, tigers, and bears are said to have a period of gestation of sixteen weeks; pumas and leopards, thirteen; our domestic cat, fifty-six days, or just half the time of the bigger felidæ; a bitch goes about sixty days; a mare, 350 days; a giraffe, 420 days; an elephant, 600; whilst an opossum is stated to go only eighteen days.

scramble, and then a rest; a period of quick breathing and then a pause. They feed much in the same way: the monkey's creed is "a little and often."

On May 27th, 1878, two brown bears were noticed to be very ill in the bear-pit. They were both violently convulsed at times; from the similarity of their symptoms, and the suddenness of the attack, there could be very little question that they had been *poisoned*. One died in a few hours. Its body and extremities were extremely rigid; the ears and the tail especially were so. I really believe that the rigid tail would have supported the extended weight of the animal. An analysis made later on of the viscera and their contents revealed, as had been anticipated, the presence of a very large quantity of strychnia. It was always suspected that a man, who had been connected with the Gardens, had poisoned these unoffending creatures; but proof of guilt could not be brought home to him. The other bear recovered only partially, then relapsed, and lingered on in a thin and miserable condition until August 17th—eighty-one days. The *post-mortem* examination showed a number of tumours on the liver, of the nature of which I have no records. The state may have been connected with chronic poisoning; but I know of no analogous cases in the human subject, although there are instances in which recovery did not occur for a month or six weeks after the strychnia had been taken. Shortly after the deaths of these two bears, another died, about five years old, and the *post-mortem* examination showed that it had stricture and invagination of the descending colon not far from the rectum. I need hardly say that no attempt at abdominal section had been made during life. Possibly the surgeons of the future may perform an "Ursine" operation; if they do, they will surely revive some Brunonian theories of "excitement!"

On May 2nd, 1889, a female llama, ten years old, died of hemorrhage from the right jugular vein. The male animal, in a savage connubial mood, had bitten her in the neck and opened the jugular vein. At least a quart of blood had been lost, when the keeper, with the aid of Mr. Munro Smith—whose kind aid has often been rendered to these helpless animals,—stanched the bleeding, and for an hour or two all went well, but then the keeper, with kind but injudicious intentions, gave the animal some oil-cake. The act of mastication and swallowing doubtless disturbed the clot, for fresh hemorrhage came on, and the animal shortly died from exhaustion. The lungs were much diseased, the right one was solidified. The male was the son of the victim, so here we have a case of matricide and wife murder combined in one.

On February 16th, 1889, the lion Jupiter, whose birth I have chronicled, and who had grown up meanwhile a worthy and very fine representative of his father, Hannibal I., had a claw on the fore-paw extracted. His health had always been very good; but for several months it had been observed that he walked about his den with a limp, which was caused by an in-growing claw. The felidæ have the power of extending and withdrawing their claws, as most of us know who have had much to do with kittens and cats. It seemed, so far as we could observe, that a claw in the left fore-paw could not be sheathed, and that it was growing into the flesh and gradually laming the animal. Possibly in their wild state the rough treatment their claws must necessarily get prevents such an accident; or it may happen—who knows? Perhaps Androclus gained the friendship of his lion by extracting a claw and not a thorn! The animal seemed to be getting worse and worse, dropped his shoulder, and was evidently often in pain. Something must be done, as time, usually the great healer and remedier, seemed inoperative here. Chloroform was out of the question: the attempts to give these animals anæsthetics have been failures and worse. The irritating and superfluous growth must be extracted, and after various "connings," there seemed to be but one plan; viz., the "cramp cage." So the operation was fixed for the next morning, and there was a fair attendance of interested spectators. The first part was getting the lion out of his den into the travelling cage, and then attaching the cramp cage to one end of it. The details are interesting; but time is precious. Let it suffice that the animal was got into the "cramp." He didn't like his quarters, and showed that even within the comparatively small dimensions he could turn round and so evade any efforts to get hold of his claw. Planks of deal, one foot broad by one-and-a-half inches thick, were then put into the cage to limit the space. The animal was fairly furious before; but now came such a display of rage, that no one who did not see it could imagine it. He fought for dear life, as he thought. Plank after plank was seized and ripped up like so much match-wood, and it seemed as if the iron bars and plates, strong as they were, would not contain the infuriated beast. His mouth bled, and he broke a tooth. Several of the keepers stood on the top of the cage to prevent it from being overturned, and some of the spectators took refuge by quietly withdrawing from the scene. At length, by putting in plank after plank, above and behind, the poor brute was brought to bay, and, to save himself from his very constrained position, pushed out his paws through the bars of the cage. "Now 's your time," I said. Blunsden immediately seized the offending claw with a pair of strong carpenter's pincers; the grip was good. The animal helped in the operation by trying his best to get his paw free, and the claw came away. It had grown into the flesh at least half an inch, most likely more; and here I can show you the very thing. In half-an-hour afterwards the creature had quite calmed down: he seemed then to have comprehended the rationale of the operation, and he gave me the conviction that if he had had to undergo a repetition he would have been a mild consenting party. The operation was completely and permanently successful.

The account of the operation got into the papers, and several of them made their comments; and amongst them the *Standard* of February 20th gave a long editorial, and raised the question of whether the necesssity for operations arose amongst wild beasts in their natural state, and also of what became of the bones of dead animals, for they were so seldom and so scantily found by hunters and travellers. I must not diverge into this question; but I can offer one explanation. We had a hyæna in the Zoo for many years, which would grind up and consume the hardest bones which were given to it. We have had other beasts with in-growing claws, and only a few months ago one of our present leopards, old Jerry, was put in the cage, and underwent an operation of having his claws pared with very little difficulty or trouble to himself.

On July 1st, 1889, a lioness, rather rickety, died of rupture of the uterus, in the right cornu. One cub had been born, for it was found in the den afterwards, and the other lay in the vagina partly extruded. The mother had been in labour five days, which must have been a very unusual time for a carnivorous or any other animal. A year or so after this, a puma died under similar circumstances. The specimen of the ruptured uterus is in the museum of the Medical School. Somewhat akin to these cases, an interesting one occurred last year.

On Saturday, May 6th, I received a message at 8.30 a.m. that an Arabian gazelle had been in labour some hours, but even with the assistance of the keeper could not produce the "kid." Examination showed that the passage was all right, but the kid was very large. I went back home and procured a pair of small midwifery forceps. Traction with these seemed to advance matters nothing; so, with the advice of Mr. Munro Smith, we determined upon abdominal section—a Cæsarian operation, may I style it? Chloroform was given, which the gentle creature took well. A very large but well-formed kid, already dead, was taken away, the wounds were carefully washed antiseptically and stitched with cat-gut; then the patient's abdomen was smoothly bandaged, and the animal slung up in a sack, with slits in it for the legs to pass through; the feet were tied together to prevent any attempts at struggling, and so obviate any tendency of the stitches in the wounds getting torn out. She went on well for some hours, and in the evening, about 10 o'clock, she was left in charge of the keepers, one of whom was to remain with her all night. She had taken a little nourishent, milk and gruel, and nibbled a little bit of hay, but at four a.m., after a slight shivering fit, when the man very feelingly threw his coat over her, she passed away. Subsequent examination showed that scarcely a stitch in the wounds was disturbed; there had been no hemorrhage, and there was no peritonitis. Many of these animals bear shock very badly indeed. She defied the arts of surgery, they were not for her, and in the night her spirit had perchance gone back to the blue hills of Persia or the lands of Arabia the Happy!

In relation to chloroform, the following case has points of interest. A favourite cockatoo had been placed in the bird-house by a lady because it was becoming blind, and she hoped something could be done to lessen its sad affliction. Examination showed that it had cataract. Mr. Cross very kindly examined the eye, and an operation was determined upon. I gave the chloroform; a piece of lint with half-a-drachm upon it was applied to the head of the bird, which could have had only an infinitesimal whiff, when it fluttered its wings and died. Either the heart, which had responded again and again to the caresses of an over-indulgent mistress, would not be cajoled by the wiles of an anæsthetic, or its life-span coincided with the time of the administration; for birds often drop down dead from their perch.

If they won't bear shock well, animals do not refrain, nevertheless, from occasionally fighting to the death. Raccoons usually seem peaceful enough together, but every now and then —whether from bad temper or a species of insanity—they have very serious frays; and about three years ago two of them must have had a terrible pitched battle one night, for when the house was opened the following morning, one of the males was discovered with a hole in the left side of its abdomen, through which protruded so much small intestine, mesentery, and midriff, that the only course to pursue was to give it a speedy despatch. Lately two female badgers fought most desperately; one was killed at once, and the other, awfully mauled, died in a day or two. About this period a young black panther, which had been born and reared in the Gardens (a very unusual event in this country), ailed for some time and died. The *post-mortem* examination gave evidence of a rupture of the liver near the gall-bladder, with effusion of bile into the peritoneal cavity. The animal was very fond of leaping about, and the supposition was that in doing so it had ruptured the liver.

On Friday afternoon, June 2nd, 1893, the larger of two Polar bears died. They had been denizens of the Gardens for nearly twelve years, having arrived in a very "cubby" condition in November, 1881, when in consequence of the ice-floes coming down far south the whaling boats had captured a good number, and Polar bears were rather at a discount. The deceased had been seen to eat her dinner as usual and with apparent avidity, when suddenly she rolled over in a fit, threw up her legs, and then, turning over and over, fell into the bath in an insensible state. The death was attributed to apoplexy; but the verdict was not verified *post mortem.* The weather was very hot, and the stuffer could not keep the head long enough for me to examine it.

The second of these Polar bears was shot on April 10th this year, and if humanity had had its proper influence—but the delay was mine—it would have ended its days sooner, and been saved the terrible sufferings I fear it endured. I think there can be no question that animals in their natural state are more mercifully dealt with than in captivity. As soon as an animal ceases to be able to procure its food—when it cannot work for its living—its life must only be a question of time. In captivity we feed them, or supply them with food. They may occasionally recover, which they would not have done in their native state; but very often we prolong the agony for them. Let us hope they do not suffer as acutely as we think they do. This bear had been falling off for some time—I dare say for Polar bears it had reached a good age—but cod-liver oil, brown bread and butter, and other delicacies had probably prolonged its existence; but it got worse and worse, looked ill and wretched, and either could not pass water properly, or else had a distended bladder, with dribbling of urine. I think the day it was killed I never saw agony of pain so depicted in any animal. It seemed maddened with suffering. We determined to have it shot, and came to the conclusion that a good charge of heavy shot discharged close to the animal would be a more effective way than by bullet. A second gun was provided in case of need, but the first shot at the distance of a foot just behind the right shoulder soon settled her. The *post-mortem* showed that the pulmonary artery had been torn through and caused extensive hemorrhage into the lungs. The bladder had ruptured, and the urine had passed out into the abdominal cavity. All the tissues seemed soft and rotten. The kidneys were large and divided into twenty or thirty lobules.

Some years ago a young lion, four and a-half months old, was found dead in its cage. For three or four days it had been ailing; its breathing was very quick, and it took no food, merely lapping a little water. A *post-mortem* examination made seven hours afterwards revealed a pericardium full of semi-purulent fluid, of the consistence of gruel and coloured by admixture with a little blood. Probably it had had pericarditis, and the inflammation had gone on to the suppurative stage.

It is possible that the disease might have been pyæmic in origin, for about this time a female cheetah, which had been in the Gardens about two years, died after about a month's illness, and after its death we found an empyema on each side, more especially the right one. The pus was of a corn-flour custard consistence, and on the right base was becoming organised into membrane. Both lungs were much compressed; the liver was congested and softened, but did not display any purulent foci; the kidneys were congested, and the skin had an unusual yellow tinge. The illness commenced with sickness and diarrhœa, followed by much wasting, with destruction of the right eye from sloughing of the cornea.

The felidæ are very prone to inflammation of the lungs. Almost every *post-mortem* examination upon them gives evidence of this, either as a recent condition or as having occurred at a more remote period. On July 10th, 1889, a male cheetah died of inflammation of both lungs, more especially the right one, and then a few days afterwards a female died; and I have several other records.

Whilst on the subject of empyema, I may mention the case of a Mona monkey which died in November, 1891, after an illness of rather more than two months. It was paraplegic nearly the whole of the time, and dragged its legs after it in a very ludicrous manner. It fed well, and except for the paralysis seemed fairly happy and comfortable. It had a swelling on the right side just below the ribs. I had the monkey taken out of the cage, and examined this swelling very carefully, and came to the conclusion that it was a fatty tumour and harmless. The *post-mortem* examination showed that an error had been made in diagnosis, for the swelling turned out to be a collection of pus in connection with an empyema of the right pleura. It is possible that puncturing the tumour, in the first instance, might have cured the paraplegia and the empyema. I say *might*, because monkeys are very bad patients; it is almost impossible to keep any kind of dressing or application upon them.

Monkeys are very liable to chest affections; and there is no question that we have lost a great many—some ordinary specimens, and some rare and of considerable value, as chimpanzees and ourangs—from tuberculous disease of the lungs. They seem very prone to pleurisy, and adhesions are frequently found, with and without tubercular masses in the lungs; but actual cavities from ulceration do not seem very frequent. The condition of two female chimpanzees was interesting, but very distressing; and I feel sure the insanitary condition of their house at that time had much to do with their illness. Whether they were related in blood, I am not sure; but they certainly were like sisters in affliction—and in affection, too. The melancholy solicitude which they showed for each other was really remarkable. The elder one, probably between two and three years old, died first, after an illness of some weeks' duration; she was seriously ill for about six weeks. At first she had troublesome diarrhœa, and then cough with rapid wasting succeeded. Her appetite became very bad—no food, fruit, or prepared milk seemed to entice her. Sometimes she would eat a few bananas; and for several days before her death took nothing else. Topsie, her younger companion—at this time in good condition,—used to watch her, and cuddle up to her in the most human and kindly manner; and when the cough was troublesome, Topsie would look deeply and appealingly distressed, as much as to say, "Can't you do anything for my friend?" The end came at last, on the 21st July, 1889; and the *post-mortem* examination showed much solidification of the right lung, enlargement of the bronchial glands, and the presence of tubercle bacilli. In rather less than two months afterwards (September 9th) Topsie herself succumbed, after having been ill for little more than a month. She coughed and wheezed very much, very like a little old woman; she had only slight diarrhœa.

I have many more particulars about the results of the autopsies, but I must not dwell on them. Let me, however, emphasise this point, that we lost in the course of a few years a great many monkeys, and they nearly all had undoubted symptoms of tubercular disease. Monkeys seem particularly obnoxious to the tubercle bacilli; I should say more so than any other animals I have noticed. There is no doubt that the monkey-house became very unhealthy, and the excessive mortality was only stayed by putting the inmates elsewhere, and thoroughly disinfecting it with sulphur. This procedure has been carried out from time to time with most satisfactory results.

Some years ago we used to possess two very fine giraffes: but now it is impossible to procure giraffes; in consequence, it is stated, of the disturbances in the Soudan, they cannot be got down to the coasts. Very large sums of money have been offered for them by zoological gardens abroad, but without results. One of our giraffes suffered from what appeared to be chronic rheumatism of the joints, chiefly those of the fore-legs; and he would hobble about in a very gouty, ludicrous manner. He was sent off in exchange for some other animals, and then died shortly after. The other lived for only a few months afterwards; dying after a short illness from congestion of the lungs, which were somewhat emphysematous, and contained some old cheesy-looking nodules. There were two small uric-acid calculi in the left kidney; the liver was soft, and the stomach greatly distended with food, by which the diaphragm was pushed up, thus impeding the action of the heart and lungs.

On several occasions this distension of the stomach has been found, arising from the practice of feeding the animals with dainties, &c., which they would not have in their natural state, and which they are doubtless, when in this weakly condition, better without. It may interest some of you to know that, absurdly long as the necks of giraffes are, they have only seven cervical vertebræ--they look as if they might have forty. These animals are becoming very scarce; and I question very much whether there is a living one now throughout the British Isles or on the Continent.

Whilst on this subject of stomach distension, it seems very appropriate to relate the following cases:—

It is not at all an uncommon thing to find in the stomach of seals quantities, even large quantities, of stones, gravel, and pieces of rocks, rounded off, which have evidently been in the stomach for a very long time. This condition is found in the sea-lions, or Cape seals, to a more marked extent than in ordinary seals. I have the notes of a case where ten pints of stones, weighing twenty-two pounds, were found. The explanation of the reason does not seem very clear. The stones are doubtless swallowed; but for what purpose? The tradition amongst the seal hunters is this—that when the seals get very fat, they cannot sink themselves in the deeper water with the same facility as when they were thinner, and so they take in the stones as ballast; and the stomachs with their strange contents are locally known as "ballast bags." The theory is a very pretty one, almost romantic; but I don't think we can accept it. I collected a good deal of information about this subject some years ago, and embodied the results in a paper I had the honour of reading before the Bristol Naturalists' Society. I think that probably there is a physiological reason. Last year, in April, a very fine Cape ostrich, the survivor of two we had in the Gardens, began to ail very seriously. He had been falling off in appetite and flesh for some time, and, in spite of every care and attention, died on April 10th. The gizzard and stomach were enormously distended, the contents consisting of about eight or nine pounds of cabbage leaves, young grass, Indian corn (which had been boiled), remnants of brass buttons, buckles and safety-pins, pieces of glass and metal, a pen-holder and two pocket-handkerchiefs, with a tell-tale name upon one, a memorandum book with a druggist's label upon it: such things, however, and pieces of money are usually found in these birds' stomachs, and consequently they may be naturally regarded as objects for use in digestion. These evidently were not the main cause of the dire dyspepsia the poor bird had; oh, no! this was the thing—the remains of a Prayer Book, with the thirty-nine articles, undigested!

Tumours occur amongst the denizens of the Gardens; but they are not common. In February, 1890, died the last of several Crimean geese. It was considered to be the oldest inhabitant of the Gardens, having been there since about 1856 or 1857. The feathers of these birds always looked as if they had been ruffled the wrong way, curled something like Japanese chrysanthemum flowers. The abdominal cavity contained a large fibroid mass of at least one pound in weight, and apparently connected with the mesentery. It certainty was not a fatty liver; for the bird was very thin, and looked as if it had long ago consumed all such material as fat.

Some months afterwards (December 5th, 1891), Mr. Budgett, of Stoke Bishop, gave us an old mare to be slaughtered for the carnivora. On opening the abdomen, an enormous tumour (fibroid) was found connected with the uterus; and this is in the museum of the Medical School, I believe.

The oldest inhabitant of the Gardens now is the griffin vulture. For years this bird has had an exostosis on the right foot. It does not get any larger; but it cripples the bird a little, and makes it hobble rather than hop about.

Towards the end of last year, a black constrictor snake—Bascanion constrictor—was noticed to have a swelling on its body a few inches from the tail, and which had gradually increased in size, and was evidently tormenting the reptile, because instead of being very lively, it began to move about carefully and cautiously, and twined itself round a dead branch in its den with great circumspection and avoided pressure upon the swelling. So, on January 24th last, I got the keeper (Keeler) to assist me. He very adroitly, with the aid of a duster, seized the snake at the back of its neck, and brought it out of the cage. The tumour was situated close to the cloaca; it was as large as a small walnut, very tense, fluctuating, and certainly very painful on pressure. I had no instrument with me except a knife, so I used a blade of this, and with it opened a kind of fæcal abscess, as very offensive matter spurted out. Great relief followed; but after a few days it was evident the mischief was not eradicated; so on February 12th a more careful operation was performed, when some fishy-smelling matter was evacuated, and then a small tent of boracic lint was placed in the wound. Eventually the wound closed. The snake has cast its skin several times since, and although I can make out a small swelling, I think the cure is a radical one.

Whilst on the subject of snakes, and especially those of the python kind, which have the reputation of being able to exercise great powers of fascination over their victims, I may say that I have watched these snakes feed on a great many occasions, and I have come to the conclusion that they have no "fascination power" whatever. I have seen ducks and fowls placed in the pythons' cages (they have a strange preference for ducks), and except for the consternation that may affect any creature when suddenly placed in new surroundings, the intended victims could not well be less disturbed in their behaviour, if the inmate of the cage had been one of their own kind instead of a stony-eyed huge snake. By-and-by they settle quietly down; and I have seen ducks and fowls actually perched upon the coiled-up body of the serpent, and rabbits hopping about in the most unconcerned manner. Of course this condition of things presupposes that the snake is not very voracious, otherwise he would probably make a dash and a great coiling-up of his prey at once. But we will conclude he is not in this humour—what happens? I have seen the snake push out his head, and a duck peck at it—nay, even induce the snake to beat a retreat from the provoking beak. And often and often in the morning when the keeper has come again, there has been no murder committed; but it may be that three or four ducks or fowls, or a rabbit or two, have entirely disappeared between closing time and the morning. I need hardly explain that the snakes are always fed after the houses have been closed to the public. What usually happens is this: the snake and its victims fraternise, touching each other every now and again, apparently on good terms; when, without any change so far as observation goes, the snake suddenly, as rapidly as the twinkle of an eye, seizes its victim and encoils it, and then after a short time commences the slow process of gorging itself. Imagine a person on apparently very friendly terms with you, talking to you and engaging your attention, and then suddenly, without any evident change of mind, striking a dagger, which had been concealed in the sleeve, into your heart or back. This is practically what the snake does, and it is the most subtle act I have ever seen: it is devilish subtlety, but it is not fascination.[1]

One word more about their feeding. Pythons certainly are very irregular feeders, and will go a much longer time without food than one would imagine. One large python celebrated Christmas-eve, 1888, by taking a duck; its next meal was on the following June 21st, when it devoured another duck, and then afterwards fed regularly about every week for some time. Another python lived for eighteen months without food; but then it died. Signor Succi and Dr. Tanner, and other celebrated fasters, may bow their heads to the snakes.

I have come across various worms from unexpected sources. A python would hardly be regarded as a host for them, yet one of our pythons last year passed two ascarides lumbricoides and a large portion of a tænia, which I have not yet been able to specialise. It is neither solium nor medio-canellata. The common English snakes not unfrequently have passed lumbrici; but one might have thought the very strong digestive juices of the python, which can dissolve the feathers of ducks and fowls and the skin of a rabbit with its fur, would have proved too much for tænia development.

On April 20th, 1891, a female rhea (American ostrich) died, after falling off in health for some time; and in each pleural cavity was a nest containing a large number of long cord-like worms—one measuring two feet one inch—and which at the time I considered were lumbrici, but which I now believe are really filariæ, the Guinea worms.

On September 1st, 1879, a very fine male lion, General, Hannibal's son, died after a short illness. The *post-mortem* exami-

[1] Since delivering this address, I have seen Hagenbeck, the celebrated menagerie-keeper of Hamburg, who is constantly receiving snakes from all parts of the world. He is a very acute observer, and he ridicules the "fascination" theory.

nation made a few hours afterwards showed a large quantity, several pints, of sanguineo-serous fluid in the peritoneal cavity, an intensely congested appearance of the peritoneum itself, which was almost black with venous engorgement, and there was a very little lymph deposit. The serous coat of the stomach was congested in patches; but the walls were thick and firm. On opening the viscus, there was a small quantity of greenish-looking fluid, and some partly-digested food; but from every fold of the mucous membrane rose up, in hydra-looking fashion, numbers of round worms, from one inch to three inches in length. I never saw such a sight of its kind: there must have been at least a hundred. The duodenum contained a few; and I thought possibly the peritonitis had been set up by some escaped ones, but careful examination did not confirm this.

On November 9th, 1889, a female leopard, which had been born in the Gardens, died when it was ten months old. It had always been rather weakly. The *post-mortem* examination showed a large number of small lumbrici in the stomach. Many of the animals have suffered at times from ascarides lumbricoides, and santonine has been given with very good results.

On May 28th, 1893, Hannibal II. died. He was born in the Gardens, and passed his life of sixteen years within an area of about twenty yards from his birth-spot. He had always been remarkably healthy, and was probably the most magnificent black-maned lion ever seen in Europe. He is perhaps almost immortalised by having been the subject of a very fine and excellent photograph by Gambier Bolton. For several weeks before his death he had been ailing, took less and less food, lapped water occasionally, became miserable and mangy-looking, and was fast losing his glorious mane. The *post-mortem* examination showed a very remarkable absence of fat—he had used it all, perhaps, in lieu of food—congested liver and lungs, and some cysts in the liver, containing clear fluid, but no hydatids. Senile decay was my verdict.

Then on January 6th this year (1894) old Jupiter died. We have seen his birth, his claw-extracting, and now we come to the conclusion of him. He was born, and he died, in the lion-house. He had been a healthy animal upon the whole; but had had attacks of indisposition at various times. For the last several years he and the stump-tail lioness had occupied the same den—except at meal-times, when they were always separated—and they went by the names of Darby and Joan. She still survives, and is now older than he was, and she still looks a very green and hearty widow, especially when her fifty-six cubs, which she has had, are taken into the reckoning. Her coat is a splendid fawn colour, and I do not see why she should not live many more years. Lions used to be kept in the Tower of London, and one named Pompey is said to have lived there seventy years. I cannot believe it. Joan looks as if she might continue, as I said, for some years; but she is not likely to reach thirty years of age. But to return to old Jupiter. He grew worse and worse, and became shabbier and shabbier-looking. At last he was condemned by the Committee to be shot. The execution of the sentence was delayed, and one Saturday night he anticipated his despatch, which was to have been on the Monday, by dying in his den. The *post-mortem* examination gave evidence of very shrunken lungs, with carnified bases; enlargement of the liver, which weighed $9\frac{1}{2}$ lbs. and contained cysts with hydatid contents; enlarged thyroid, and intestinal mischief. The spleen and kidneys were normal; the heart was large and flabby. Mr. Morton very kindly examined the viscera for me, and his notes are: "In the lower part of the ileum, just above the ileo-cæcal valve, there is a bossed outgrowth into the lumen of the bowel, infiltrating its wall. It does not greatly obstruct the lumen. The ileum above the growth is enormously thickened, but not dilated. Sections of the growth showed in some areas the structure of columnar epithelioma, and in the thickened wall above much round-cell infiltration and fibroid growth."

We may have massive patients and "claimants" upon our attention sometimes; but I can assure you when you come to treat an elephant, you feel very small indeed. Our lady one in the Zoo is now thirty-two years of age, and is supposed not yet to have reached maturity. She has been in the Society's possession for about twenty-seven years, and she has very seldom ailed. About the end of June, 1890, however, she began to be very unwell, whether from cold or some sexual disturbance it is difficult to say; but her skin became very wrinkled, and her trunk hung down in the most listless manner. She almost entirely refused food and even water. The ears and trunk felt very cold, and her eyes were dull and the conjunctivæ congested. What could be the matter? What medicine ought she to have? Calomel, jalap, or both? Ammonia or other stimulants? The treatment was really "expectant," and slowly she recovered; but she had several wretched days. She would lie at times on her side, with her head resting on the straw, and she would move her tail about in a very funny and despondent manner. Boiled rice and sugar, and gruel and treacle (their turtle soup), which elephants like much, were both objectionable, and the first food she took with any relish was composed of buns, gingerbread, and jam tarts. Then she got on to young carrots, especially their green tops, and afterwards completely recovered. Just lately she has had a kind of whitlow or fascial abscess on a toe of the right fore-foot. We think she must have injured the part, in the first place, by striking it against some spikes placed to guard the doors from her kicks. Hot fomentation and bran and linseed poultices relieved her, and encouraged suppuration, which took place. She walked about in a very lame manner; but now she seems almost well. I thought it would be interesting and novel to listen to the heart-beat of this huge quadruped. A horse's heart beats about thirty or forty times in a minute, and I expected an elephant's might be even slower. I expected to hear and feel a regular sledge-hammer pulsation; but, to my astonishment and disappointment, place my stethoscope where I liked—on the front of the chest, on either side, beneath, between, and behind the fore legs, and in the axillæ—not a sound could I hear, nor could I feel a pulsebeat anywhere. I don't know the anatomy of an elephant, and it may be that the heart is placed deeply and centrally in the chest cavity. The behaviour of the patient was very amusing. Of course her keeper was present. She was very quiet and docile; but she evinced a curious nervousness. A vibratory noise emanated from her trunk, and every now and then she would very lightly flick the stethoscope with the end of her trunk. I can assure you I never felt so ridiculous in my life as when I stood by her huge body and placed a pigmy instrument upon her thick skin.

I have trespassed upon your patience, I am sure, too long. If I have not imparted to you much information, I hope I have not altogether failed to interest you. The great German poet, Goethe, said, "When I observe the luminous progress and expansion of Natural Science in modern times, I seem to myself like a traveller going eastwards at dawn, and gazing at the growing light with joy, but also with impatience; looking forward with longing to the advent of the full and final light, but, nevertheless, having to turn away his eyes when the sun appeared, unable to bear the splendour he had awaited with so much desire." We are all waiting for this full fruition of light; and if I have advanced you and myself this evening one iota towards this desirable prospect, my effort, which has been accompanied with much pleasure to myself, and which you have received with such patient endurance, will not have been in vain.

